# Case Report: Elevated CPK, an indicator of idiopathic inflammatory myopathy?

**DOI:** 10.12688/f1000research.7681.1

**Published:** 2016-02-12

**Authors:** Hina N. Khan, Usman Jilani, Shitij Arora

**Affiliations:** 1 Internal Medicine, Montefiore Medical Center of the Albert Einstein College of Medicine, Bronx, USA; 2 New York Institute of Technology College of Osteopathic Medicine, Long Island, USA

**Keywords:** Idiopathic inflammatory myopathy, Polymyositis, Myasthenia

## Abstract

Polymyositis is a rare disease with incidence rates at about 1 per 100,000 people annually. In this case report we will review a case of proximal muscle weakness with an elevated creatine phosphokinase that was initially misdiagnosed twice as rhabdomyolysis. Therefore, emphasizing that idiopathic inflammatory myopathy is a potential cause of myasthenia that must be considered in the differential. The case will also describe the current treatment and treatment response in polymyositis.

## Introduction

When a clinician is confronted with a case of muscle weakness in the setting of a severely elevated creatine phosphokinase by probability alone, the most likely culprit is rhabdomyolysis. Incidence rates of rhabdomyolysis are about four to five-fold that of polymyositis ^1^. This is reflected in the abundance of medical literature citing cases of rhabdomyolysis. This case report highlights a patient who presented with symptoms of an elevated creatine kinase and myasthenia, but was eventually diagnosed with an idiopathic inflammatory myopathy. Idiopathic inflammatory myopathies have only rarely been described in patients as the etiology of these diseases is poorly understood. Mechanisms of action including cellular-mediated cytoxic mechanisms in polymyositis, to a complement-mediated vasculopathy of the small vessels in muscle tissues in dermatomyositis, to a primarily macrophage driven degeneration in immune-mediated necrotizing myopathy have all been ascribed as the pathophysiology behind idiopathic inflammatory myopathies ^2^. Currently, there is an understanding that both genetic and environmental factors play a role in unmasking an inflammatory myopathy ^3^. Our case reviews why the presence of proximal muscle weakness, an elevated creatinine phosphokinase, and systemic clues should raise the clinician’s suspicion for these poorly understand idiopathic inflammatory myopathies.

## Case

A 64-year-old man presented with two weeks of progressive proximal muscle weakness causing him difficulty ambulating, combing his hair, and raising himself from a seated position. He also reported dysphagia to solids over the past week. The patient was recently admitted twice to an outside hospital for an elevated creatine phosphokinase, and diagnosed with “rhabdomyolysis.” Past medical history was significant for poorly controlled diabetes mellitus. On exam, vitals were within normal limits. Cardiovascular exam revealed regular rate and rhythm with no extra heart sounds and lung exam revealed clear lungs on auscultation. On neurologic exam, patient was awake, alert, and oriented. Cranial nerves II through XII were grossly intact. There was proximal muscle weakness to 4/5 bilaterally in the shoulders and hips, but preserved strength distally. Sensation to light touch remained intact. Hemoglobin was 10.5 g/dL with a normal MCV, WBC were 3.8 K/uL, and platelets were 246 K/uL. Basic metabolic panel revealed a sodium of 141 mEq/L, a potassium of 4.8 mEq/L, and a creatine of 0.90 mg/dl. Transaminases including AST and ALT were 65 and 36 U/L, respectively. CPK was 2705 U/L. ESR and CRP were within normal limits and RF, anti-Ro/La, anti-Jo1, anti-RNP, anti-centromere, anti-Scl-70, and anti-dsDNA were all negative. An MRI of the right arm revealed prominent edema involving the deltoids and an MRI of the bilateral thighs revealed myositis with no discrete collections. An EMG showed evidence of irritative myopathy affecting the bilateral deltoids. A muscle biopsy of the right deltoid was negative for inflammation but presumed to not be representative. He was started on oral Prednisone 60mg daily with resolution of symptoms including weakness and pain and normalization of creatine kinase within one month of starting therapy.

## Discussion

Myasthenia, or simply muscle weakness, is a common admitting diagnosis in the inpatient setting with a broad differential. However, in the setting of an elevated CPK the differential diagnosis for myasthenia is narrowed to rhabdomyolysis or a myopathy. While acute exertional rhabdomyolysis can be diagnosed by history, the syndrome of idiopathic inflammatory myopathies must be differentiated from myopathies caused by infections, toxins, paraneoplastic syndrome, and endocrinopathies. Idiopathic inflammatory myopathy encompasses systemic rheumatic diseases including polymyositis and dermatomyositis. Our patient met three out of the four criteria for polymyositis per the American College of Rheumatology including symmetric proximal muscle weakness, elevation of skeletal muscle enzymes, and an abnormal EMG showing polyphasic, short, small motor unit potentials, fibrillation potentials, positive sharp waves, and repetitive high-frequency discharges, suggesting a probable diagnosis of polymyositis. The final criteria needed for definitive diagnosis is an abnormal muscle biopsy with histopathologic findings of degeneration, regeneration, necrosis, and interstitial mononuclear infiltrates, in this case, muscle biopsy was presumed not representative as his anterior right deltoid had been sampled as opposed to the posterior or lateral deltoid which is typically higher yield. However, several epidemiologic features make this a particularly difficult diagnosis in this patient. Foremost, polymyositis is a rare disease with incidence rates occurring in about 1 per 100,000 people annually ^4– 7^. Additionally, polymyositis is seen twice as commonly in women than in men. Furthermore, although polymyositis can occur at any age, it typically peaks in the 30–50 year age range ^7, 8^, and our patient had a later age of onset. This case also highlights the low sensitivity of serologic tests including anti-Jo-1, anti-Scl-70, and anti-RNP which are prevalent in 21%, 6%, and 5% of patients with polymyositis, respectively ^9^.

However, when considering a case of myasthenia with an elevated CPK, some key features can be used to differentiate polymyositis from other diagnoses. These include chronicity of symptoms, presentation of symmetric proximal muscle weakness, and presence of bulbar features, which can be seen in polymyositis, dermatomyositis, or inclusion body disease. Other systemic signs including rash seen in dermatomyositis, or Raynaud Phenomenon seen in scleroderma or CREST can also aid in correctly arriving at a diagnosis. Steroids remain the cornerstone of treatment in polymyositis although lack of response as high as 40% has been reported. Such a high non-response rate may partly be due to misdiagnosis of inclusion body myositis as polymyositis. Studies are ongoing to identify specific mechanisms for steroid resistance with higher expression of proinflammatory molecule “granulysin” in CD8+ T cells and glucocorticoid receptor polymorphisms as possible explanations ^10, 11^. In conclusion, polymyositis is a rare disease with several epidemiologic features and serologic markers which are neither sensitive nor specific, but key history and physical exam findings can help arrive at a diagnosis of polymyositis when confronted with a case of myasthenia and an elevated CPK, and thus aid in promptly initiating appropriate therapy.

## Consent

Written informed consent for publication of their clinical details was obtained from the patient.
