# Supplementary material for: Case Report: Elevated CPK, an indicator of idiopathic inflammatory myopathy?
Source: F1000Res. 2016 Feb 12;5:164. [Version 1] doi: 10.12688/f1000research.7681.1 (PMC4965691; doi:10.12688/f1000research.7681.1)
Supplement: Peer review history

# Peer review: Referee response for version 1

**DOI:** 10.5256/f1000research.8271.r15273

**Published:** 2016-07-28

**Authors:** Xenofon Baraliakos

**Affiliations:** 1 Department of Rheumatology, Ruhr University Bochum, Herne, Germany

This is an interesting case of a differential diagnosis for inflammatory myopathy. I only have minor suggestions or open questions.

- Was ESR only tested once? What about previous examinations which might have shown increased values? In daily practice we ask for such results when we see these patients (e.g. from previous GP consultations) and it would be interesting to see if the patient never has had increased inflammatory markers, which would be a bit unusual.
- Should there be an MR image of the pathologic finding, it would be nice to add it to this report, if the space allows.
- Did you ever consider doing a second biopsy for the case that the right area has been missed? If not, it would be worth it to add a sentence in the Discussion about the role of repeated biopsies in such cases.
- The patient had reported to have poorly controlled diabetes mellitus. What happened to that condition after 1 month of high-doses of cortisone and how did you deal with it? Again, 1-2 sentences in the Discussion would be informative in order to think of how to solve this comorbidity.

I have read this submission. I believe that I have an appropriate level of expertise to confirm that it is of an acceptable scientific standard.

# Peer review: Referee response for version 1

**DOI:** 10.5256/f1000research.8271.r13251

**Published:** 2016-05-05

**Authors:** Martin Aringer

**Affiliations:** 1 Department of Medicine III, University Medical Center Carl Gustav Carus, TU Dresden, Dresden, 01307, Germany

This is an educational case of a 64 year old male who presented with a fairly typical picture of a few weeks of proximal muscle weakness, difficulties swallowing, increased AST and very high CPK levels, but was initially misdiagnosed as having rhabdomyolysis. The absence of a usual trigger of rhabdomyolysis, the lack of rapid improvement and the pharyngeal involvement were early clues that the latter differential diagnosis was not correct. A muscle biopsy was unfortunately negative, and the authors found no specific autoantibodies, so that the diagnosis is not certain. The clinical picture, the MRI findings, and the improvement under glucocorticoids, which usually takes at least two weeks, nevertheless support that this patient has polymyositis. The only unusual finding is the normal ESR. In addition, the authors should have tested for antinuclear antibodies (ANA) by indirect immunofluorescence on HEp2 cells, where both ANA and cytoplasmic fluorescence would have given further clues. Many of the patients with anti-synthetase antibodies do have additional symptoms, ranging from mild Raynaud’s and mild arthritis to mechanics’ hand and early signs of interstitial lung disease. In addition to anti-Jo-1 antibodies, several other anti-synthetase antibodies can be tested for today. Some patients will have malignancies underlying new onset polymyositis or, more often dermatomyositis.

Recommendations for (small) changes:

1. understood (instead of understand) in the last sentence of the Introduction.
2. in the setting of very high CPK values… (instead of elevated CPK values) second sentence of the Discussion.
3. The final criterion (instead of criteria) in the middle of the first paragraph of the Discussion; a reference should also be added there.
4. Raynaud’s (instead of Raynaud) and systemic sclerosis (instead of scleroderma or CREST) in the second paragraph of the Discussion   

I have read this submission. I believe that I have an appropriate level of expertise to confirm that it is of an acceptable scientific standard.
